# Deep learning for the rapid automatic quantification and characterization of rotator cuff muscle degeneration from shoulder CT datasets

**DOI:** 10.1007/s00330-020-07070-7

**Published:** 2020-07-22

**Authors:** Elham Taghizadeh, Oskar Truffer, Fabio Becce, Sylvain Eminian, Stacey Gidoin, Alexandre Terrier, Alain Farron, Philippe Büchler

**Affiliations:** 1 ARTORG Center for Biomedical Engineering Research, University of Bern, Freiburgstrasse 3, CH-3010 Bern, Switzerland; 2 Department of Diagnostic and Interventional Radiology, Lausanne University Hospital and University of Lausanne, Lausanne, Switzerland; 3 Laboratory of Biomechanical Orthopedics, Ecole Polytechnique Fédérale de Lausanne, Lausanne, Switzerland; 4 Service of Orthopedics and Traumatology, Lausanne University Hospital and University of Lausanne, Lausanne, Switzerland

**Keywords:** Computed tomography, Deep learning, Muscle atrophy, Rotator cuff, Sarcopenia

## Abstract

**Objectives:**

This study aimed at developing a convolutional neural network (CNN) able to automatically quantify and characterize the level of degeneration of rotator cuff (RC) muscles from shoulder CT images including muscle atrophy and fatty infiltration.

**Methods:**

One hundred three shoulder CT scans from 95 patients with primary glenohumeral osteoarthritis undergoing anatomical total shoulder arthroplasty were retrospectively retrieved. Three independent radiologists manually segmented the premorbid boundaries of all four RC muscles on standardized sagittal-oblique CT sections. This premorbid muscle segmentation was further automatically predicted using a CNN. Automatically predicted premorbid segmentations were then used to quantify the ratio of muscle atrophy, fatty infiltration, secondary bone formation, and overall muscle degeneration. These muscle parameters were compared with measures obtained manually by human raters.

**Results:**

Average Dice similarity coefficients for muscle segmentations obtained automatically with the CNN (88% ± 9%) and manually by human raters (89% ± 6%) were comparable. No significant differences were observed for the subscapularis, supraspinatus, and teres minor muscles (*p* > 0.120), whereas Dice coefficients of the automatic segmentation were significantly higher for the infraspinatus (*p* < 0.012). The automatic approach was able to provide good–very good estimates of muscle atrophy (*R*^2^ = 0.87), fatty infiltration (*R*^2^ = 0.91), and overall muscle degeneration (*R*^2^ = 0.91). However, CNN-derived segmentations showed a higher variability in quantifying secondary bone formation (*R*^2^ = 0.61) than human raters (*R*^2^ = 0.87).

**Conclusions:**

Deep learning provides a rapid and reliable automatic quantification of RC muscle atrophy, fatty infiltration, and overall muscle degeneration directly from preoperative shoulder CT scans of osteoarthritic patients, with an accuracy comparable with that of human raters.

**Key Points:**

*• Deep learning can not only segment RC muscles currently available in CT images but also learn their pre-existing locations and shapes from invariant anatomical structures visible on CT sections.*

*• Our automatic method is able to provide a rapid and reliable quantification of RC muscle atrophy and fatty infiltration from conventional shoulder CT scans.*

*• The accuracy of our automatic quantitative technique is comparable with that of human raters.*

## Introduction

Knowledge of the status of rotator cuff (RC) muscles is key in various shoulder disorders, not only RC tendon tears [1] but also glenohumeral osteoarthritis [2, 3]. In particular, muscle degeneration parameters such as fatty infiltration and atrophy influence surgical decision-making and overall patient management [4, 5]. Although magnetic resonance imaging (MRI) offers higher contrast resolution for the evaluation of soft tissues, computed tomography (CT) still allows for the detailed quantitative analysis of muscles, distinguishing between muscle, fat, and bone tissues using specific Hounsfield unit (HU) thresholds [6–8]. Furthermore, CT is widely available, fast, and well accepted by patients, and this examination is increasingly being used in the imaging evaluation of glenohumeral osteoarthritis and preoperative planning of shoulder arthroplasty [9–11].

In clinical practice, the status of RC muscles is currently assessed using qualitative and/or semi-quantitative methods, most notably Thomazeau’s occupation ratio [12] or Zanetti’s tangent sign [13] for supraspinatus muscle atrophy and the Goutallier classification for fatty infiltration [1], which are all fast and easy to use but also only moderately accurate and/or reliable [14, 15]. More robust and accurate quantitative CT techniques have been developed but have not yet established themselves in increasingly busy clinical workflows, mainly because of time constraints [6, 7]. Automation of such techniques would make them clinically viable and could further promote the use of CT as the one-stop-shop imaging prior to shoulder replacement surgery. In recent years, deep learning has emerged as a very effective classification technique, which has been applied with great success to medical image segmentation, including muscle segmentation in CT datasets [16–19], and detection of large rotator cuff tears from conventional shoulder radiographs [20]. However, to the best of our knowledge, this technique has yet to be evaluated for the prediction of the premorbid muscle boundaries, which are not distinctly and readily identifiable in the images.

Therefore, this study aimed at developing and evaluating the performance of a CNN able to automatically assess RC muscles from shoulder CT images. RC muscles were assessed by quantifying their various degeneration parameters, most notably muscle atrophy and fatty infiltration. Unlike traditional segmentation tasks, the neural network must in this particular case not only segment the structures currently available in the images but also learn the pre-existing locations, shapes, and boundaries of RC muscles from invariant anatomical structures visible on CT sections.

## Materials and methods

### Dataset

Our dataset consisted of all consecutive preoperative shoulder CT scans of patients treated with anatomical total shoulder arthroplasty for primary glenohumeral osteoarthritis between January 2002 and December 2014 (*n* = 172). Patients with CT arthrography and/or metal artifacts (*n* = 43) were excluded, as well as patients with non-overlapping CT sections and/or reconstructed axial CT images thicker than 1.25 mm and/or using sharp kernels only (*n* = 26). The resulting study population consisted of 103 shoulder CT scans from 95 different patients (62 females and 33 males; mean age, 70.5 years; age range, 36–89 years; mean body mass index (BMI), 27.1; BMI range, 17.7–39.4; 62 right and 41 left shoulders). The most relevant raw shoulder anatomical characteristics from this dataset are provided in Table 1. Furthermore, 12 (12%) shoulders had secondary bone formations (glenoid osteophytes, secondary osteochondromas, and/or heterotopic ossifications), while 37 (36%) cases showed glenohumeral joint effusion with or without synovitis, and 5 (5%) cases exhibited subacromial bursitis. No patient had soft tissue masses in the shoulder such as lipomas. This study was approved by the institutional ethics committee (CER-VD protocol 505/15).

**Table 1:** Relevant raw shoulder anatomical characteristics of the CT dataset used in this study

Supraspinatus muscle with substantial atrophy			Supraspinatus muscle with substantial fatty infiltration	Glenoids with substantial retroversion	
Occupation ratio < 50%	Negative tangent sign	Both occupation ratio < 50% & negative tangent sign	Goutallier 3 & 4	Walch B2 & B3	Walch C
*n* = 8 (8%)	*n* = 5 (5%)	*n* = 8 (8%)	*n* = 0 (0%)	*n* = 27 (26%)	*n* = 5 (5%)

Shoulder CT scans were part of the routine preoperative planning for these patients and performed on several multi-detector row (from 4 to 64, all from GE Healthcare) CT scanners using standardized data acquisition settings. Relevant image reconstruction parameters were as follows: display field of view, 15 × 15–25 × 25 cm (pixel size, 0.29 × 0.29–0.49 × 0.49 mm); section thickness, 0.63–1.25 mm; section interval, 0.3–1 mm; and smooth convolution kernel.

**Fig. 1:**
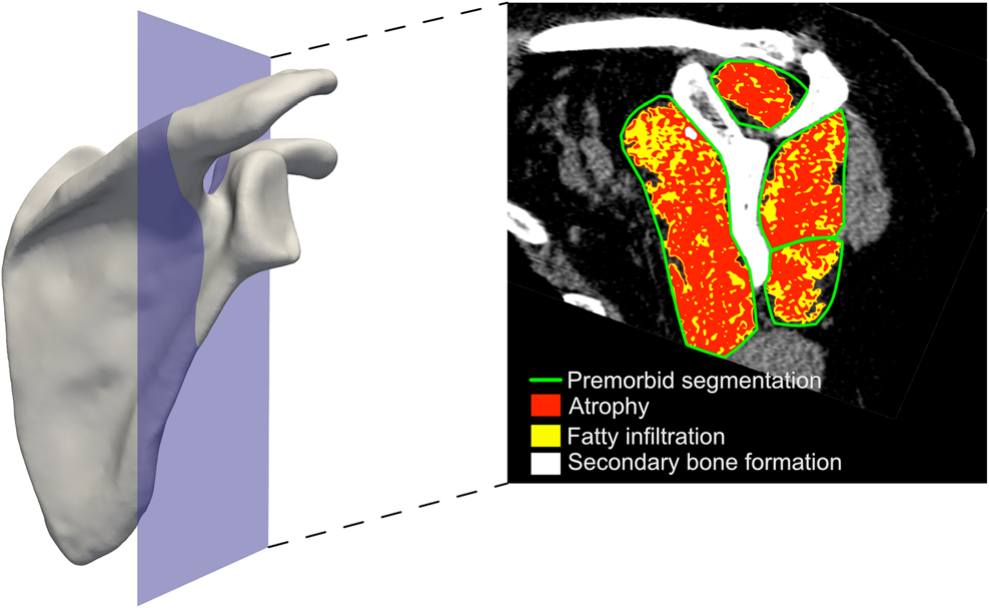
The segmentation of RC muscles was performed on a standardized sagittal-oblique CT section (left). First, the premorbid boundaries of all four RC muscles were identified on this section, manually by human raters and automatically by the deep learning algorithm (right, green delineation). Then, automatic threshold-based image processing was used to quantify and characterize the cross-sectional area of each remaining/atrophied RC muscle (right, red), with its amount of fatty infiltration (right, yellow) and secondary bone formation (right, white)

The identification of the premorbid shape of all four RC muscles was performed on a standardized sagittal-oblique CT image (Fig. 1) [7]. This reconstructed CT section was defined as the plane perpendicular to the scapular axis and passing through the spinoglenoid notch. The best-fitting line along the supraspinatus groove was used to determine the scapular axis [21, 22]. All four RC muscles (supraspinatus (SS), subscapularis (SC), infraspinatus (IS), and teres minor (TM)) from each case were manually segmented by three independent musculoskeletal radiologists with varying levels of training (from 2 to 13 years of experience).

### Deep learning

The variability in the training dataset was augmented by introducing on all images with varying degrees of scaling and rotation. This was also deemed useful to make the method applicable to differently formatted images. Images were scaled by a random factor comprised between + 20 and − 20% and combined with a rotation by a random angle between + 90° and − 90°. This data augmentation resulted in a tenfold increase in sample size for a total of 3090 segmented CT images per RC muscle (103 cases × tenfold augmentation × 3 raters). All images were resampled to a resolution of 512 × 512 pixels prior to deep learning.

**Fig. 2:**
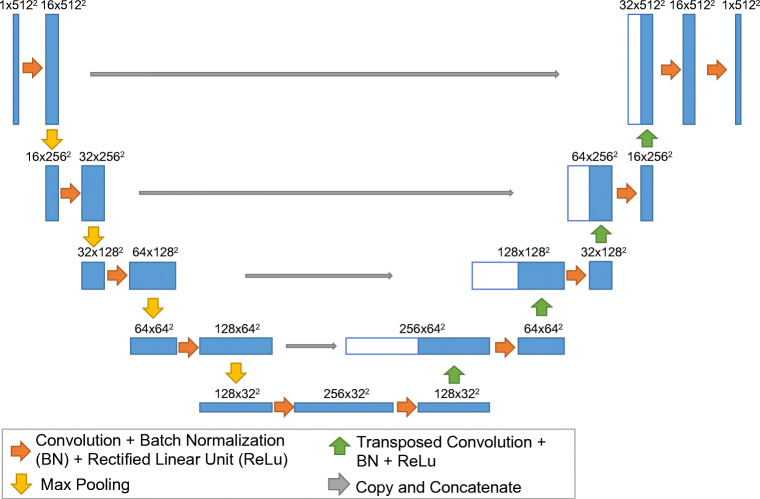
Architecture of the convolutional neural network used in this study. The main difference compared with the original U-Net proposed by Ronneberger et al [23] is that only one convolution layer is used after each max pooling. In addition, batch normalization was applied after each convolution layer

A CNN following a traditional U-Net architecture was used in this study [23]. The neural network consisted of a repetition of alternating convolution layers followed by maximum pooling layers. After four repetitions of the combined convolution and downsampling layers, the 512 × 512 pixels input image resulted in a 32 × 32 data representation with 512 channels (Fig. 2). We modified the original U-Net architecture by including a single convolution layer after each up-/downsampling layer. In addition, our network included a batch normalization for each convolution layer [24] (Fig. 2).

A fivefold cross-validation was used to iteratively train and test the neural network. The training dataset was divided into five subsamples of equal sizes, each containing 618 segmentations per muscle. One subsample was iteratively selected for testing, while the remaining four subsamples were used to train the CNN. This approach resulted in training 20 different networks (4 muscles × fivefold cross-validations) to provide a fully automatic segmentation of the entire CT dataset. The random separation of data performed for the cross-validation step ensured that the network was agnostic to the validation set. During the training phase, a validation split of 1% of samples was used to determine the best-performing network configuration.

After segmentation of premorbid RC muscles by the CNN, all CT images were upscaled from 512 × 512 pixels to their original resolution of 1024 × 1024 pixels. Segmented images were then further processed by identifying each current muscle as the largest connected component in the CNN-segmented image output, and by filling any holes in the segmentation.

### Analysis

Automatic segmentations were evaluated against a reference segmentation that was generated for each RC muscle by aggregating the three manual segmentations using the simultaneous truth and performance level estimation (STAPLE) expectation-maximization algorithm [25]. STAPLE computes a probabilistic estimate of the true segmentation from a collection of delineations executed by trained human raters. Automatic segmentations were compared with the corresponding STAPLE reference segmentations using two metrics: Dice coefficients and Hausdorff distances. The Dice similarity coefficient quantifies the similarity of two samples using an index ranging between 0 (no segmentation overlap) and 1 (perfect segmentation overlap). The Hausdorff distance is the greatest distance between a point on the surface of a segmentation and the closest point on the corresponding one. Similarly, inter-rater reliability was assessed by calculating Dice coefficients and Hausdorff distances between each of the three different pairs of human raters. Paired Student *t* tests were used to compare automatic segmentation with inter-rater variability. Results were considered statistically significant at *p* < 0.05.

Furthermore, manually and automatically predicted premorbid RC muscle segmentations were both used to determine the ratio of muscle atrophy, fatty infiltration, secondary bone formation (including osteophytes, secondary osteochondromas, and heterotopic ossifications), and overall muscle degeneration, according to the method proposed by Terrier et al [7]. Briefly, CT numbers in each pixel were used to determine the type of tissue (muscle, fat, or bone). First, a lower threshold of − 29 HU was applied within the premorbid segmentation (S) of each muscle. Holes of the resulting segmentation were filled and islands removed to determine the outer boundary of the residual/atrophied muscle (Sa). Within this surface Sa, fatty infiltration (Si) was quantified as the surface below − 29 HU and secondary bone formation (So) as the surface above 166 HU. Based on these measurements, we determined atrophy (Ra = Sa/S), fatty infiltration (Ri = Si/S), secondary bone formation (Ro = So/S), and overall muscle degeneration (Rd = (Sa + Si + So)/S). The overall muscle degeneration ratio has a value of 0 when the muscle is fully healthy, and 1 when completely degenerated.

Linear regressions were used to quantify the relationship between the muscle degeneration parameters obtained using manual and automatic segmentations. Regression analysis was further used to evaluate the variability of muscle degeneration quantification between human raters, and impact of patient BMI on the quality of the automatic segmentation (together with Pearson correlation coefficients). The *R*-squared values and the slope of the regressions were used as a measure of performance.

## Results

**Fig. 3:**
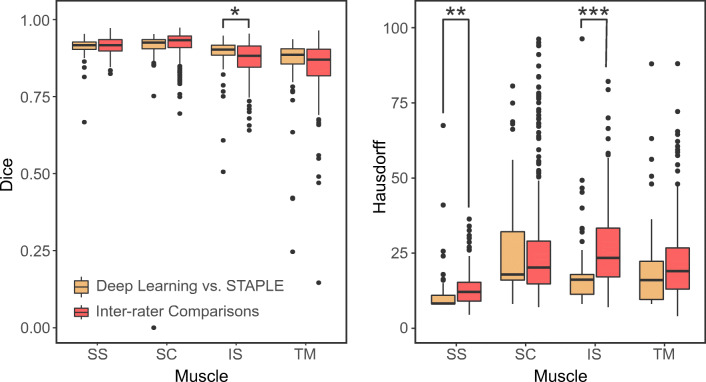
Dice similarity coefficients (left) and Hausdorff distances (right) between the automatic deep learning and STAPLE reference manual segmentations, and compared to Dice coefficients between manual segmentations from different human raters. Note that inter-rater evaluations contain three times more data points (309 evaluations) than the evaluation of the deep learning segmentation (103 evaluations). This difference results from the multiple evaluations necessary to evaluate the different possible combinations of human raters. Statistical differences are indicated by one star (*) if *p* < 0.05, two stars (**) if *p* < 0.01, and three stars (***) for *p* < 0.001

Manual premorbid RC muscle segmentations showed a high inter-rater reliability with an average Dice coefficient of 89% ± 6% when considering all muscles together (Table 2 and Fig. 3). The TM muscle had the lowest Dice coefficient between human raters (85% ± 8%), while the SS and SC muscles showed the highest inter-rater reliability with a Dice coefficient of 92% ± 3% (Fig. 3).

**Table 2:** Overview of the results obtained automatically with the deep learning algorithm and manually by human raters for the segmentation of the premorbid boundaries of all four RC muscles, and for the subsequent quantification of the degeneration of each individual muscle. “DL-STAPLE” stands for the correlation between results obtained by deep learning (DL) and the simultaneous truth and performance level estimation (STAPLE) true segmentation, while “Inter-rater” reports results obtained by human segmentations. Means and standard deviations are reported for Dice coefficients and Hausdorff distances. Slopes and *R*^2^ of linear correlations between DL predictions and the STAPLE reference model, as well as between different human raters, are also reported for muscle atrophy, fatty infiltration, secondary bone formation, and overall muscle degeneration for each RC muscle. For Dice coefficients and Hausdorff distances, statistical differences are indicated by one star (*) if *p* < 0.05, two stars (**) if *p* < 0.01, and three stars (***) for *p* < 0.001

				Atrophy		Fatty infiltration		2nd bone formation		Overall degeneration	
		Dice	Hausdorff	Slope	*R*^2^	Slope	*R*^2^	Slope	*R*^2^	Slope	*R*^2^
SS	DL-STAPLE	0.91 ± 0.03	10.7 ± 7.2**	0.96	0.95	0.93	0.97	0.89	0.77	0.96	0.96
	Inter-rater	0.92 ± 0.03	13.0 ± 5.3**	0.97	0.90	0.95	0.92	0.95	0.78	0.97	0.93
SC	DL-STAPLE	0.91 ± 0.09	28.5 ± 34.4	0.73	0.82	0.96	0.86	0.26	0.18	0.87	0.92
	Inter-rater	0.92 ± 0.04	28.3 ± 22.6	0.91	0.82	0.98	0.89	1.07	0.83	0.96	0.91
IS	DL-STAPLE	0.89 ± 0.06*	19.4 ± 17.4***	0.94	0.93	0.96	0.93	0.47	0.45	0.96	0.97
	Inter-rater	0.87 ± 0.05*	26.5 ± 13.3***	0.93	0.93	1.00	0.93	0.88	0.64	0.97	0.97
TM	DL-STAPLE	0.86 ± 0.10	18.6 ± 15.6	0.86	0.71	0.91	0.84	0.35	0.10	0.89	0.77
	Inter-rater	0.85 ± 0.08	21.9 ± 14.1	0.94	0.73	0.94	0.84	0.32	0.11	0.95	0.78

**Fig. 4:**
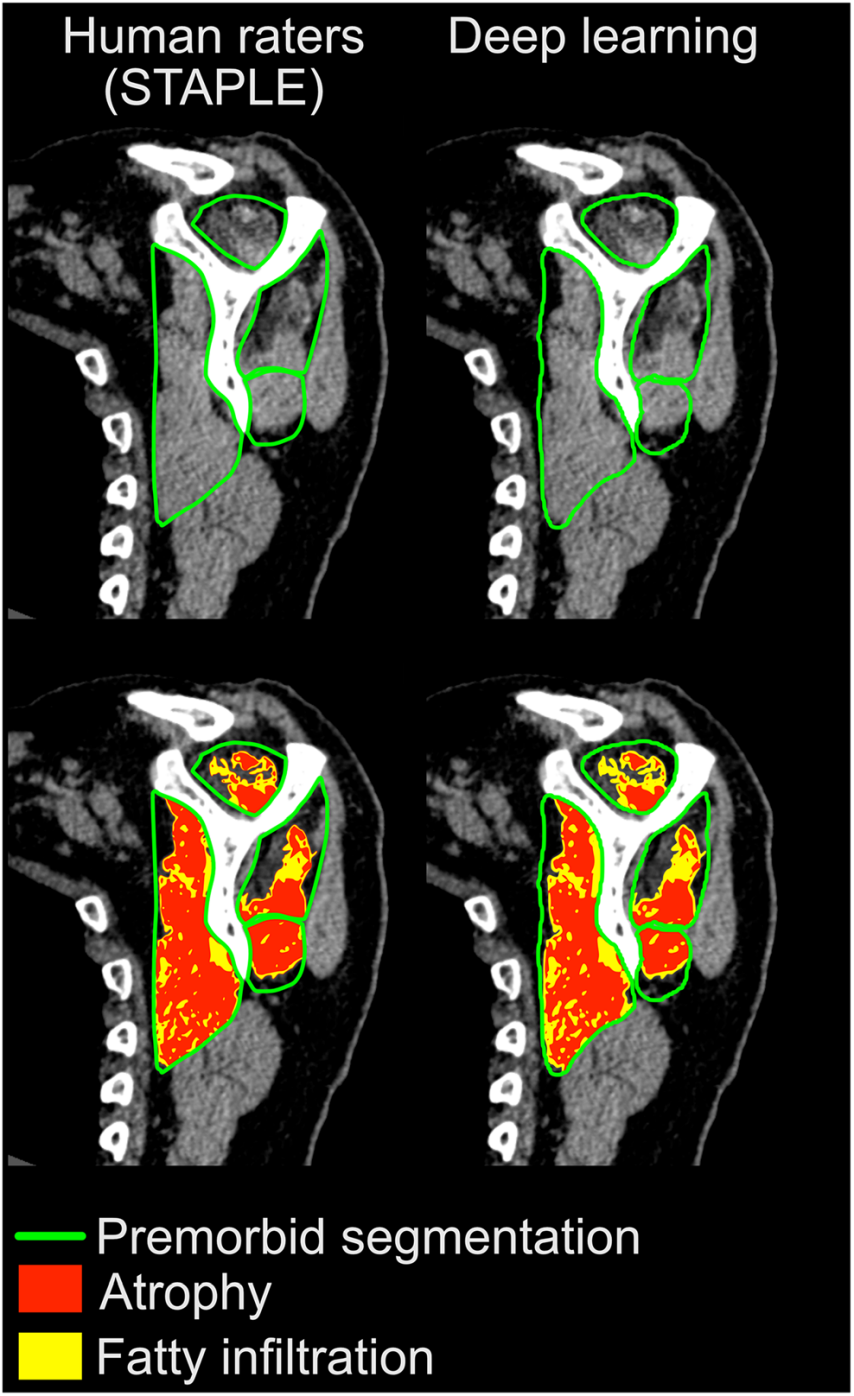
Representative sagittal-oblique CT images showing the steps of muscle segmentation (top) and quantification and characterization of RC muscle degeneration (bottom) in a selected osteoarthritic patient. Results obtained manually by human raters (STAPLE reference) for each individual RC muscle are shown on the left, compared with deep learning quantification on the right

Similar results were obtained with the automatic segmentation approach; overall, the average Dice coefficient was 88% ± 9% when comparing the outcome of the CNN with the corresponding STAPLE reference segmentations (Fig. 4). No significant differences were found between Dice coefficients for segmentations obtained with the CNN and human raters for the following three muscles: SC (*p* = 0.120), SS (*p* = 0.341), and TM (*p* = 0.398). However, the neural network yielded a significantly higher Dice coefficient for the IS muscle (*p* = 0.012). Nevertheless, even for this muscle, the difference in the Dice coefficient between the automatic and manual segmentations remained less than 2% (Table 2 and Fig. 3).

The Hausdorff distance between the CNN (automatic) and STAPLE (manual) reference segmentations was smaller than the distance between human raters (Table 2 and Fig. 3). CNN segmentations yielded significantly lower Hausdorff distances for the SS (*p* = 0.004) and IS (*p* < 0.001) muscles. No significant differences were found for the other two muscles (SC, *p* = 0.96; TM, *p* = 0.06).

**Fig. 5:**
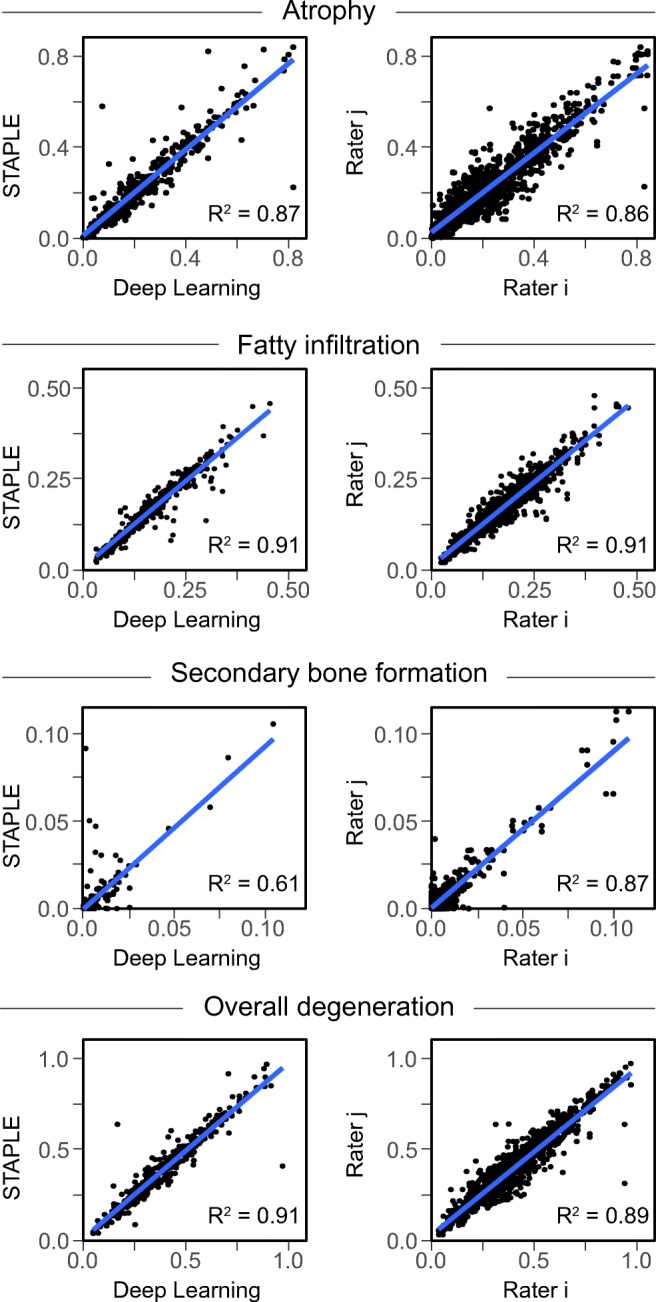
Linear correlations for muscle atrophy, fatty infiltration, secondary bone formation, and overall muscle degeneration between automatic deep learning predictions and manual STAPLE reference model (left), as well as between different human raters (right). Except for secondary bone formation, the *R*^2^ values are equal or higher for the deep learning approach

**Fig. 6:**
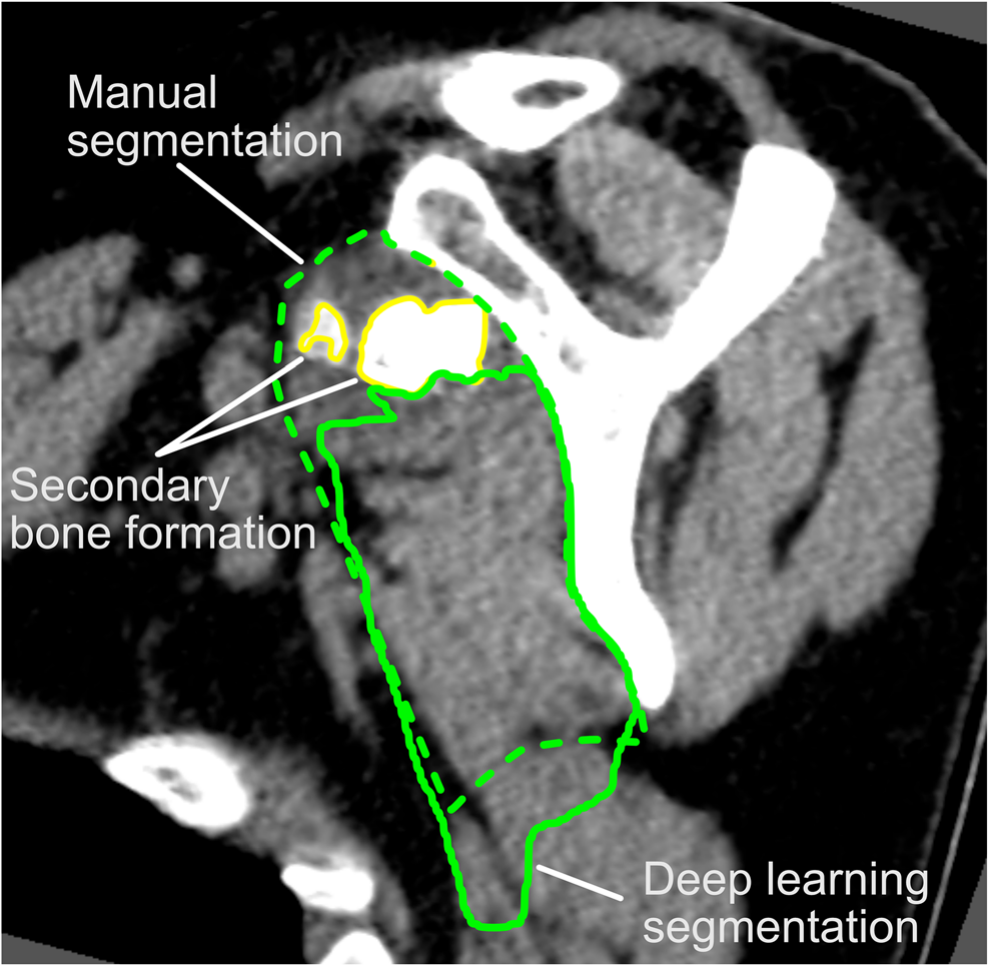
Representative sagittal-oblique CT image showing a rare case of severe secondary bone formation in a patient with secondary osteochondromatosis of the glenohumeral joint. In this setting, the deep learning algorithm failed to capture the premorbid boundaries of the SC muscle

The automatic approach was able to provide good–very good estimates of muscle atrophy (*R*^2^ = 0.87), fatty infiltration (*R*^2^ = 0.91), and overall muscle degeneration (*R*^2^ = 0.91), with an average regression slope of 0.95 ± 0.05 (range, 0.86–1.02) (Fig. 5). These relationships were comparable with the results achieved by human raters. However, segmentations by the CNN showed a higher variability in the quantification of secondary bone formation (*R*^2^ = 0.61) than human raters (*R*^2^ = 0.87). In fact, some of the automatic segmentations incorrectly included small parts of the scapular bone adjacent to RC muscles, or failed to delineate the boundaries of RC muscles when large secondary bone formations were located in close proximity to the scapula (Fig. 6). Again, the TM muscle was more difficult to predict both for the CNN and human raters, with coefficients of determination *R*^2^ as low as 0.7 for muscle atrophy (Table 2).

**Fig. 7:**
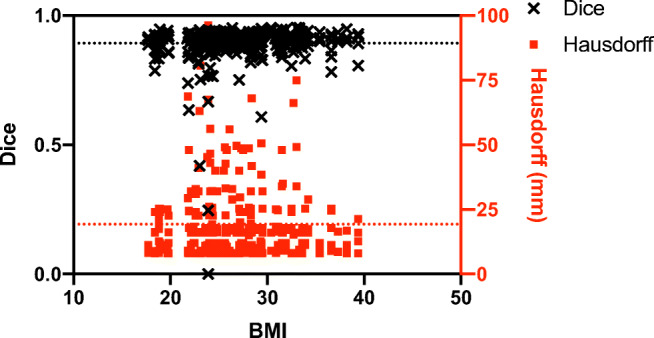
Scatter plot of Dice coefficients and Hausdorff distances as a function of patient BMI showing that the quality of the automatic segmentation was not significantly affected by patient BMI and its related CT image quality

Patient BMI, and related CT image quality, had no impact on the quality of the automatic segmentation. The regression slopes between BMI and Dice coefficient and BMI and Hausdorff distance were both not significantly different from 0 (Fig. 7). In addition, for each of the four RC muscles, Pearson correlation coefficients were very weak for both the Dice coefficient (|*r*| ≤ 0.15) and Hausdorff distance (|*r*| ≤ 0.11).

On average, human raters delineated a single case consisting of four RC muscles in about 2–3 min. While the training of the CNN took approximately 100 h of calculation, the delineation of the four muscles took less than 1 s per case.

## Discussion

This study aimed to assess whether deep learning could rapidly and automatically predict RC muscle degeneration from shoulder CT scans with acceptable accuracy and reliability, particularly for the diagnosis and planning prior to total shoulder arthroplasty. We developed and validated a new method to quantify the degeneration of RC muscles from shoulder CT images and compared its performance against three human raters with varying levels of experience. Convolutional neural networks were used to delineate the premorbid boundaries of each of the four RC muscles on a standardized sagittal-oblique CT section, and muscle degeneration was subsequently quantified and characterized in terms of muscle atrophy, fatty infiltration, and secondary bone formation.

Our automatic method was able to determine the premorbid locations, shapes, and boundaries of all four RC muscles with an accuracy comparable with manual segmentations. In addition, the quantitative parameters describing muscle degeneration derived from this automatic premorbid delineation were highly correlated with the results obtained by three different human raters for muscle atrophy, fatty infiltration, and overall muscle degeneration. These results indicate that this automatic quantitative technique reached a level of accuracy equivalent to human raters and provides accurate and reliable predictions, almost instantly and without fatigue.

An exception to the successful quantification of RC muscle degeneration is the assessment of the level of secondary bone formation, where the automatic quantification method failed to reproduce the results of human raters. This moderate accuracy mainly results from the difficulty in segmenting the interface between the scapula and the various RC muscles. In the case of localized bone outgrowth “inside” the muscle, the deep learning algorithm tended to follow the bone contours, while human raters realized that this “heterotopic” bone protrusion was caused by the degeneration process and should thus be included in the premorbid boundaries of the involved RC muscle. However, these few localized mis-segmentations had only a marginal impact on the overall quality of the segmentations, and no effect on the other markers of muscle degeneration, but strongly affected the quantification of secondary bone formation (also considering that it is the smallest muscle parameter in terms of cross-sectional area). Overall, our dataset included only a few (12/103, 12%) cases with secondary bone formation, which was mainly encountered in patients with advanced glenohumeral osteoarthritis. Increasing the number of cases with substantial secondary bone formation would certainly enable the CNN to improve its segmentation performance in this setting.

Automatic segmentation of the IS and SS muscles presented lower Dice coefficients and/or Hausdorff distances than human segmentations. Although this result might look counter-intuitive, two aspects can explain this behavior. First, the segmentation performance of machine learning was evaluated against STAPLE, which calculates a probabilistic estimate of the true segmentation. Therefore, if one of the human raters provides a segmentation that is very different from the other raters, his segmentation will have a lower contribution to the STAPLE estimate of the true segmentation. On the contrary, the human rater with a “poor” segmentation will have a more important effect on the inter-rater evaluation. The second explanation concerns the anatomical location and boundaries of these muscles, both of them being contained in a bony/muscular fossa (the SC muscle has a relatively wide fatty boundary anteriorly). The strong signal intensity of bone in the image can easily be detected by the neuron network, providing highly repeatable segmentations. Nevertheless, it is important to note that the differences (although statistically significant) remained numerically small.

Other methods have been proposed to evaluate RC muscle degeneration. In particular, quantifying muscle atrophy from shoulder MR images was initially proposed by Thomazeau et al [12]. This measurement technique determines the muscle occupation ratio, which is defined by the ratio between the muscle and its fossa cross-sectional areas on a sagittal-oblique section. However, this method is limited to the SS muscle and does not take into account other markers of muscle degeneration such as fatty infiltration. Goutallier et al [1] first developed a semi-quantitative grading system to assess fatty infiltration from axial CT images. This method became an accepted standard and was later transposed to the sagittal-oblique plane and adapted to MRI [8]. However, this classification remains of limited precision (when transposed numerically, stage 2 comprises fatty infiltration ranging from around 10 to 45%) and reliability, as shown by substantial intra- and inter-rater variability [14, 15, 26]. To address these issues, more robust semi-automated quantitative CT methods have been proposed [6, 7]. While such algorithms have effectively improved the reliability of image-based muscle assessment, they still require human raters to manually delineate the assumed premorbid shapes and boundaries of each RC muscle on CT or MR images, which is time-consuming and thus greatly limits the clinical applicability and spread of these approaches.

More recently, deep learning and CNN techniques have been used to provide an automatic quantification of muscle fatty infiltration in neck muscles from MR images [17] or abdominal muscles from CT datasets [27, 28]. Both studies reported good agreement between the automatic approach and human raters. However, these studies were limited to the segmentation of the current morbid muscle shape visible in the images but did not account for any degeneration processes by predicting the premorbid muscle anatomy. Therefore, such studies were unable to quantify and characterize muscle atrophy or overall degeneration.

The major limitation of our study concerns the selection of the oblique CT section used to determine the premorbid boundaries of RC muscles. This image was obtained semi-automatedly by selecting a series of well-identifiable landmarks on the surface of the scapula [21, 22]. As such, the overall assessment of RC muscle degeneration is not yet fully automatic. However, several studies have shown that automatic identification of bone landmarks is feasible, either relying on registration algorithms [29–31] or deep learning [32–34]. Moreover, the 2D automatic evaluation developed in our study could be further extended in 3D to quantify muscle degeneration in the entire CT dataset. However, the automatic identification of the oblique CT section was beyond the scope of this study, where we aimed at determining if deep neural networks were able to determine the premorbid locations, shapes, and boundaries of all four RC muscles.

Second, the dataset used in our study was limited to patients scheduled for anatomical total shoulder arthroplasty and did not include patients requiring reversed prostheses. The latter cases would exhibit higher muscle atrophy and fatty infiltration. Although the methodology developed here to predict the premorbid shape of the RC muscles is applicable to more severe cases of muscle degeneration, the model required proper training and validation for the latter patients. In addition, although some patients had glenohumeral joint effusion with/without synovitis (37/103, 36%) and/or subacromial bursitis (5/103, 5%), our initial dataset did not include any soft tissue masses such as lipomas. While joint or bursal effusion did not affect the performance of automatic segmentation, the presence of soft tissue masses would certainly have led to CNN segmentation failure, as in the case of secondary bone formations.

Third, the assessment of the method was limited to CT datasets reconstructed using smooth convolution kernels dedicated to the analysis of soft tissues. Preliminary analyses showed that quantification accuracy decreased when using sharp kernels dedicated to evaluating bone structures, mainly because of higher image noise. This limitation could certainly be overridden by training the CNN with a larger number of noisier sharp reconstructions. However, the vast majority of clinical shoulder CT scans are reconstructed using both sharp and smooth kernels.

Nevertheless, our study showed that it is now possible to provide an accurate and reliable characterization of RC muscle degeneration with a robust quantitative technique that might replace the standard-of-care qualitative or semi-quantitative methods currently being used in daily clinical practice [1, 12, 13]. In addition, the segmentation and quantification processes are automatic and can be performed almost instantly by a computer, which is significantly less than the 2–3 min required for a human observer to perform the same task manually on a dedicated workstation in an increasingly busy clinical workflow.

The novel method presented here for shoulder CT scans has the potential to be incorporated into routine diagnostic algorithms and preoperative planning to further personalize the therapeutic approach, and help select the optimal surgical technique and implant design in shoulder arthroplasty. However, further clinical validation, with a more heterogeneous and complete dataset including many comorbidities, is required to determine the clinical accuracy of this technique, and its potential impact on clinical management and outcome. With such a tool, we expect to improve the imaging assessment and classification of the patient’s shoulder morphology prior to surgery, which would impact surgical decision-making and overall patient management. This method can further be used for the rapid analysis of large patient cohorts/series in order to investigate potential associations between RC muscle degeneration and the occurrence of specific shoulder disorders, or the clinical outcome of related treatments.

## Abbreviations

BMI: Body mass index
CNN: Convolutional neural network
CT: Computed tomography
HU: Hounsfield unit
IS: Infraspinatus
MRI: Magnetic resonance imaging
RC: Rotator cuff
SC: Subscapularis
SS: Supraspinatus
STAPLE: Simultaneous truth and performance level estimation
TM: Teres minor
